# Effects of Maternal Dietary Patterns during Pregnancy on Early Childhood Growth Trajectories and Obesity Risk: The CANDLE Study

**DOI:** 10.3390/nu12020465

**Published:** 2020-02-13

**Authors:** Zunsong Hu, Frances A. Tylavsky, Mehmet Kocak, Jay H. Fowke, Joan C. Han, Robert L. Davis, Kaja Z. LeWinn, Nicole R. Bush, Sheela Sathyanarayana, Catherine J. Karr, Qi Zhao

**Affiliations:** 1 Department of Preventive Medicine, University of Tennessee Health Science Center, Memphis, TN 38163, USA; zhu24@uthsc.edu (Z.H.); ftylavsky@uthsc.edu (F.A.T.); mkocak1@uthsc.edu (M.K.); fjay@uthsc.edu (J.H.F.); 2 Departments of Pediatrics and Physiology, University of Tennessee Health Science Center, and Children’s Foundation Research Institute, Le Bonheur Children’s Hospital, Memphis, TN 38103, USA; jhan14@uthsc.edu; 3 Center for Biomedical Informatics, Department of Pediatrics, University of Tennessee Health Science Center, Memphis, TN 38103, USA; rdavis88@uthsc.edu; 4 Department of Psychiatry, University of California San Francisco, San Francisco, CA 94118, USA; kaja.lewinn@ucsf.edu; 5 Departments of Pediatrics and Psychiatry, University of California San Francisco, San Francisco, CA 94118, USA; Nicole.Bush@ucsf.edu; 6 Seattle Children’s Research Institute, Center for Child Health, Behavior, and Development, Seattle, WA 98121, USA; sheela.sathyanarayana@seattlechildrens.org; 7 Department of Pediatrics, University of Washington, Seattle, WA 98121, USA; ckarr@uw.edu; 8 Department of Environmental and Occupational Health Sciences, University of Washington, Seattle, WA 98195, USA; 9 Department of Epidemiology, University of Washington, Seattle, WA 98195, USA

**Keywords:** childhood obesity, fast food, growth trajectory, maternal dietary pattern, pregnancy

## Abstract

We investigated the associations between maternal dietary patterns during pregnancy and early childhood growth trajectories and overweight/obesity risk in offspring. Maternal diet was assessed using a food frequency questionnaire during the second trimester, and dietary patterns were derived by reduced rank regression. The associations between maternal dietary pattern scores and body mass index (BMI) trajectories from birth to age four (rising-high, moderate, and low BMI trajectories) as well as overweight/obesity risk at age four were analyzed (*n* = 1257). Two maternal dietary patterns were identified. The fast food pattern included a higher intake of fried chicken and fish, fruit juices, mayonnaise, and sugar-sweetened beverages, while the processed food pattern included a higher intake of dairy, salad dressing, processed meat, and cold breakfast cereal. Women with greater adherence to the fast food pattern were more likely to have children in the rising-high BMI trajectory group [OR (95% CI) = 1.32 (1.07–1.62); *p* = 0.008] or having overweight/obesity at age four [OR (95% CI) = 1.31 (1.11–1.54); *p* = 0.001]. The processed food pattern was not associated with these outcomes. The maternal dietary pattern during pregnancy represented by fried foods and sugar-sweetened beverages may contribute to rapid early childhood growth and increased risk for obesity in offspring.

## 1. Introduction

Childhood obesity continues to be a major public health concern in the US. According to national data, one-third of children and adolescents aged 2–19 years were overweight (16.6%) or obese (18.5%) in 2015–2016 [1]. Children who are overweight or obese are not only more likely to be overweight or obese as adults [2,3,4], but also have increased risk for type 2 diabetes [5,6], cardiovascular disease [7,8], and psychological or behavioral consequences, such as anxiety, depression, stress, and social withdrawal [9]. Childhood obesity has imposed a heavy economic burden on the health care system [10,11,12]. Identifying the early risk factors of childhood obesity will guide prevention efforts to reduce this burden and promote long-term health.

According to the Developmental Origins of Health and Disease (DOHaD) hypothesis, changes in the intrauterine environment at critical/sensitive periods of the developmental process could have irreversible, lifelong consequences in offspring metabolism [13]. Maternal diet during pregnancy is the primary source of energy for the fetus [14] and supplies key nutrients for fetal development [15]. Previous studies have provided some evidence that intake of individual foods or nutrients during pregnancy, such as refined grains [16] and n-6 polyunsaturated fatty acid [17], impacts childhood obesity risk. However, foods and nutrients are not consumed in isolation, but rather these components of the whole diet may form complex synergy and interaction effects [18]. Indeed, dietary pattern analysis has emerged as an alternative approach to investigate the effects of overall diet on the risk of disease, helping to deliver a clearer public health message regarding how individuals might improve their diets [18].

Very few studies have examined the associations of maternal dietary patterns during pregnancy and childhood growth and obesity risk, and results across these prior studies are inconsistent [19,20,21,22]. For example, an Irish birth cohort identified a mother’s processed food pattern, enriched with pizza, processed meat, chips, and roast potatoes, was positively associated with offspring being overweight and obese at age 5 [20]. A recent Asian study also identified a vegetable, fruit, and white rice pattern that was associated with lower offspring triceps skinfold from birth through 54 months of age [22]. In contrast, the Generation R study, a Dutch cohort, found that a vegetable, fish, and vegetable oil dietary pattern, a high-fiber cereals dietary pattern and a margarine, snacks and sugar dietary pattern were not associated with BMI, fat mass index, and risk of being overweight at age 6 after adjustment for sociodemographic and lifestyle factors [19]. However, a birth cohort of 389 mother-child pairs (91.5% nonblack and 8.5% black) in the US couldn’t identify any significant maternal dietary patterns during pregnancy associated with child growth outcomes in the first three years of life after adjustment for confounding factors [21]. The aim of this study was to identify maternal dietary patterns during pregnancy which were associated with offspring growth trajectories derived from the repeated measurement of child body size conducted annually from birth to age four years as well as overweight/obesity risk at age four. Furthermore, our large-scale birth cohort includes a sufficient number of black and white mother-child dyads to explore race-specific links between maternal diet and childhood growth outcomes.

## 2. Materials and Methods

### 2.1. Study Subjects

All mothers and children in this analysis were participants in the Conditions Affecting Neurocognitive Development and Learning in Early Childhood (CANDLE) study, a prospective birth cohort of mother-child dyads in Shelby County, Tennessee [23,24,25]. Briefly, 1503 healthy women aged 16–40 years and in their second trimester of a singleton pregnancy were enrolled between 2006 and 2011. Exclusion criteria included an existing chronic disease requiring medication (e.g., hypertension, diabetes, and sickle cell disease), known pregnancy complications (e.g., complete placenta previa and oligohydramnios), or plans to deliver at a nonparticipating hospital [24]. This study included 1257 CANDLE mother-child pairs with available maternal dietary data, of whom 820 mothers were black (65.2%) and 437 were white (34.8%). The CANDLE study was conducted in accordance with the Helsinki Declaration and approved by the Institutional Review Board of The University of Tennessee Health Science Center. Informed consent was given by participants 18 years or older, while assent was given by those less than 18 years and consent provided by their legally authorized representative prior to enrollment.

### 2.2. Maternal Measures

Self-administered questionnaires were used to collect sociodemographic information (age, race/ethnicity, education, insurance type, and marital status), lifestyle (cigarette smoking and alcohol use during pregnancy), parity, and medical history at enrollment. Self-reported height and weight prior to pregnancy were collected at enrollment and used to calculate pre-pregnancy BMI (pBMI) as weight (in kilograms) divided by the square of height (in meters). Gestational weight gain (GWG) was calculated by subtracting self-reported pre-pregnancy weight from maternal weight at delivery extracted from medical records. Gestational diabetes was assessed at clinical visits during second and third trimesters and at delivery. 

### 2.3. Maternal Dietary Assessment during Pregnancy

The Block Food Frequency Questionnaire (FFQ) was administered by interview at enrollment (the second trimester) to assess usual intake of 111 food and beverage items during the previous three months. The Block FFQ has been shown to be a valid and reliable instrument to rank individuals according to dietary and nutrient intake [26]. Interviewers were trained by registered dietitians and re-certified by a registered dietitian based on a taped interview every six months to estimate the frequency and quantity of intake. The FFQ was processed by NutritionQuest (Berkeley, CA, USA) to assess macro and micronutrient intake. The 111 food items were categorized into 36 pre-defined food groups that were based on similarities in nutrient composition, culinary, or consumption habits. An overview of these predefined food groups and the corresponding food items derived from the FFQ are shown in Table S1. Energy-adjusted serving data for each of the food groups were used for dietary pattern analyses.

### 2.4. Maternal Dietary Pattern Analysis

We used the reduced rank regression (RRR) method to identify maternal dietary patterns during pregnancy [27]. The RRR method identifies linear functions of food groups (dietary patterns) explaining most of the variation in intermediate response variables, which are preselected by investigators and believed to be related to the outcome of interest [28]. Therefore, the RRR method is more likely to identify dietary patterns that are associated with outcomes of interest, rather than breaking down the study population by all the possible dietary patterns among the studied subjects. The number of dietary patterns derived by the RRR method is equal to the number of selected intermediate response variables. In this study, maternal pBMI and GWG were selected as the intermediate response variables based on the evidence of their associations with child growth trajectories and obesity risk from previous studies, including our prior study [29,30,31,32]. It was also based on the biological plausibility that maternal weight and weight gain during pregnancy might mediate the effects of maternal diet on fetal development and growth outcomes after birth. In a certain dietary pattern derived from the RRR method, a factor loading was calculated for each food group, indicating the direction and strength of correlation with the dietary pattern. Food groups with factor loadings ≥0.2 were considered the major foods associated with the dietary pattern [33,34,35,36]. We named each dietary pattern based on these major foods and their overlapping with previously reported dietary patterns to facilitate description. Once these patterns were identified, a continuous dietary pattern score was calculated by summing up the servings intake of each food group multiplied by its factor loading for each mother to indicate that participant’s adherence to a certain dietary pattern, with a higher pattern score indicating greater adherence to a dietary pattern.

### 2.5. Child Measures

Birth weight and length of the children were extracted from medical charts by research assistants [37]. The body weight and length/height were also measured at each annual visit until 4 years old using the methods guided by the National Health and Nutrition Examination Survey protocol [38]. Body weight was measured using a digital scale. Recumbent length was obtained at the year 1 visit and standing height was obtained for those 2 years or older. The sex- and age-specific BMI z-score and percentile for each child were calculated based on the World Health Organization growth standards (<2 years) and the Center for Disease Control and Prevention (CDC) growth charts (≥2 years) as recommended by CDC [39]. Three BMI z-score trajectories (rising-high, moderate, and low BMI) among the CANDLE children were identified using the latent class growth modeling approach in our previous study (Figure S1) [29]. Briefly, the rising-high BMI trajectory group started from an average birth size followed by a rapid BMI gain during the first year and stable high BMI until four years old; the moderate-BMI trajectory group characterized by an average size at birth followed by a moderate BMI z-score gain rate; and the low-BMI trajectory group exhibited relatively lower birth size and rapid BMI gain during the first year and stayed at a relatively lower but normal BMI level until four years old [29]. Childhood overweight and obesity at age 4 was defined according to CDC criteria [40]. Overweight was defined as a BMI at or above the 85th percentile and below the 95th percentile for children of the same age and sex. Obesity was defined as a BMI at or above the 95th percentile for children of the same age and sex. 

### 2.6. Child Dietary Assessment

The information of breastfeeding was collected at the 4-week home visit as well as 1- and 4-year clinic visits by asking the question “Did you breastfeed your child?”. In addition, a 24-h dietary recall for the child including food and beverage consumed was administered every 3 months from birth to 1 year and every 6 months afterword until 3 years old. All the 24-h dietary recall data have been processed using Nutrition Data System for Research software (http://www.ncc.umn.edu/products/) to yield energy, macro, and micronutrient intakes. These repeated measurements were used to generate energy intake trajectories during early childhood using the latent class growth modeling approach. Different energy intake trajectories were observed among the children of black (three trajectory groups) and white (two trajectory groups) mothers of CANDLE (Figure S2).

### 2.7. Statistical Analysis

Descriptive statistical analysis was conducted to assess clinical characteristics of the overall sample as well as according to the quartiles of the fast food dietary pattern score. Multinomial logistic regression models were used to examine the associations of mothers’ dietary pattern scores with child growth trajectories (three categories) and overweight or obesity risk at age 4. Two regression models were constructed. The first one included potential confounding factors, including maternal age, race, education, insurance type, marital status, total energy intake, alcohol intake and smoking during pregnancy, parity, and gestational diabetes and child sex. Considering maternal gestational diabetes and children’s birth weight, gestational age at birth, breastfed status, and energy intake trajectories might mediate the effects of prenatal dietary patterns on childhood outcomes, we further included these variables in the second regression model and conducted mediation analyses for these variables using the R package “mediation” [41]. The interaction terms of dietary pattern scores with maternal race were added in regression models to examine the potential differences in the associations between dietary patterns and childhood outcomes between blacks and whites. Since the fast food dietary pattern was significantly associated childhood outcomes of interest in the aforementioned methods, logistic regression models were further used to calculate the children’s ORs of being in the low BMI and rising-high BMI trajectories as well as being overweight/obese at age 4 for the 2nd, 3rd, and 4th quartiles of the fast food pattern score as compared with the lowest quartile. Both SAS (Version 9.4; SAS Institute, Cary, CA, USA) and R (Version 3.6.1; https://www.r-project.org/) were used for the data analysis. All statistical tests were 2-sided, and a *p*-value < 0.05 was considered statistically significant.

## 3. Results

### 3.1. Identified Dietary Patterns in CANDLE

Using the RRR method with maternal pBMI and GWG as intermediate response variables, we identified two maternal dietary patterns, the fast food pattern and the processed food pattern (Table 1). The fast food pattern included higher intakes of food groups such as fried chicken and fish, fruit juices, mayonnaise (as well as margarine and butter), and sugar-sweetened beverages (factor loadings ≥0.20). The processed food pattern included higher intakes of food groups such as dairy, salad dressing, processed meat, cold breakfast cereal, canned fruit, and French fries (factor loadings ≥0.20). Details of the consumption of the 36 food groups across the quartiles of the two dietary pattern scores are listed in the Tables S2 and S3.

### 3.2. Study Participant Characteristics

Study participant characteristics according to the quartiles of the fast food pattern score are shown in Table 2. With increasing adherence to the fast food pattern score, mothers were more likely to be black, single, Medicaid or Medicare-insured, and multiparous, and had less education and higher pBMI levels. Children of the mothers with greater adherence to the fast food pattern were less likely to be breastfed, but more likely to be obese at age four.

### 3.3. Associations of Prenatal Dietary Patterns with Childhood Outcomes

After adjusting for potential confounders, a higher maternal fast food pattern score was significantly associated with a greater risk of being in the rising-high BMI trajectory among children (Table 3). In addition, the maternal fast food pattern score was also positively associated with the risk of overweight and obesity in children at age 4. However, the maternal processed food pattern score was not significantly associated with these childhood outcomes. The effects of these two maternal dietary patterns during pregnancy on the studied childhood outcomes were not different by race (all P values for the interaction terms of race and dietary patterns >0.05) (Table 3). Gestational diabetes, birth weight, gestational age at birth, breastfed, and childhood energy intake trajectories did not mediate the effect of maternal fast food pattern on the childhood growth trajectories and obesity risk (Table S4). Further including these variables in the regression models didn’t significantly change the associations between maternal dietary patterns and childhood outcomes (Table 3). The fast food pattern was associated with higher intakes of fat and lower intakes of important micronutrients for fetal development (e.g., vitamin D, zinc, and folic acid) (Table S5).

**Figure 1:**
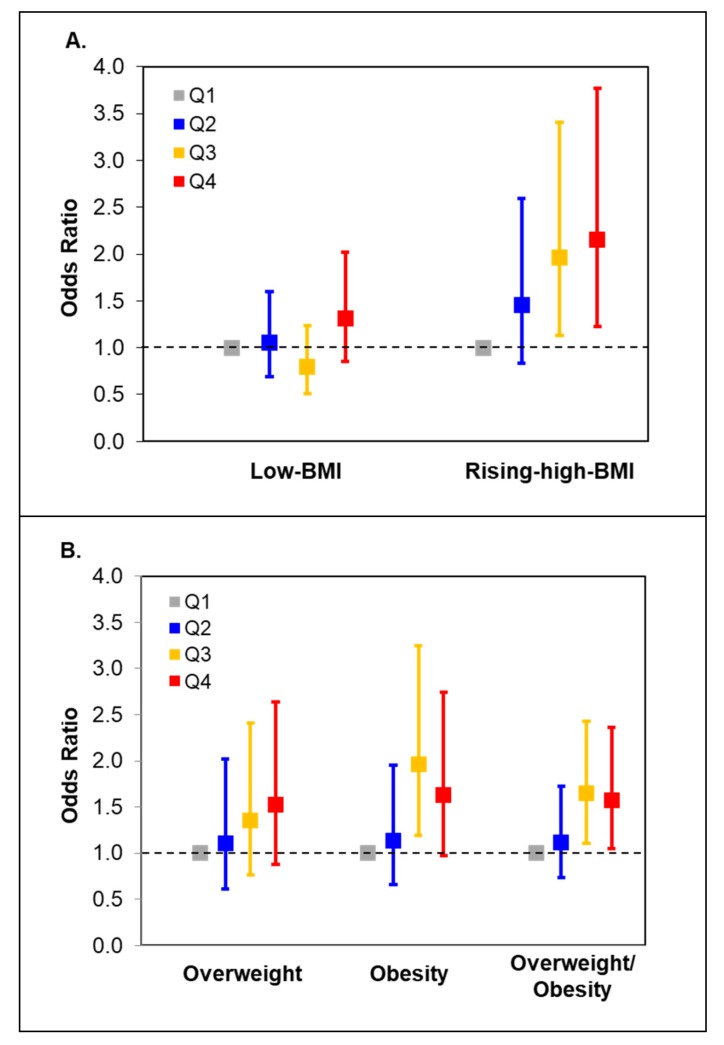
Odds ratios (95%CI) for child growth trajectories (**A**) and overweight/obesity (**B**) across maternal quartiles of the fast food pattern score.

Figure 1 shows the odds ratios (ORs) for the rising-high BMI trajectory and overweight/obesity in children according to the fast food pattern score quartiles in mothers. Compared with children of mothers who were in the lowest quartile of the fast food pattern score, those of mothers who were in the highest quartile had increased risk of being in the rising-high BMI trajectory [OR (95% CI): 2.15 (1.23–3.77); *p* = 0.008] and being overweight or obese at age four [OR (95% CI): 1.57 (1.05–2.36); *p* = 0.029]. 

## 4. Discussions

In this large-scale prospective birth cohort, we found that greater maternal adherence to a fast food dietary pattern during pregnancy was significantly associated with increased risk for child rapid growth after birth and overweight and obesity at age four. These findings further highlight the important role of maternal diet during pregnancy in child growth and obesity risk.

A limited number of studies have investigated the impact of maternal dietary patterns during pregnancy on childhood obesity, and the findings are still not conclusive [19,20,21,22]. These discrepant findings may be attributed, perhaps in part, to the variation in study populations and dietary assessment tools. Food intake varies among different populations, so dietary patterns tend to be population-specific and vary a lot among studies. In our study, we identified a fast food dietary pattern during pregnancy associated with child growth trajectories and overweight/obesity risk at age four. These findings are in line with previous studies reporting a relationship between maternal consumption of these food groups and offspring growth outcomes [42,43]. For example, the Omega study, a prospective pregnancy cohort in the US, reported that regular intake of fried fish and fried chicken was associated with an elevated risk of gestational diabetes [42], which is an established risk factor for childhood obesity [44]. In addition, the Generation R study identified a significant positive association between maternal consumptions of sugar-containing beverages and children’s BMI during early childhood [43]. Additionally, we did not observe significant associations between the maternal processed food pattern and child growth outcomes, although previous studies have shown some evidence of its impact on childhood obesity [20,21]. All the reported processed food patterns included processed meat but varied in other food items. In other words, although these dietary patterns were all called the “processed-food” pattern in different studies, the food components and contributions varied. For example, the Irish birth cohort identified a mother’s processed food pattern, enriched with pizza, chips, and roast potatoes in addition to processed meat which were different from the major food items of the processed-food pattern we identified. Dietary patterns tend to be population-specific because different populations may have different culture, eating habits, and food availability. This might explain these inconsistent findings about the processed food dietary patterns to some extent.

The mechanisms linking maternal fast food diet pattern and childhood obesity are unclear. We examined the potential mediation effects of gestational diabetes, birth outcomes (birth weight and gestational age at birth) and feeding practice (breastfeeding and childhood energy intake trajectories) during early childhood. However, we did not find that these factors mediated the effect of the maternal fast food dietary pattern on children’s weight gain and obesity risk. The poor nutrient profile of the fast food dietary pattern, which includes high fat and sugar content and low micronutrients essential during pregnancy, may have direct impacts on placental and fetal growth and metabolism, leading to adverse postnatal outcomes in offspring [40]. On the other hand, the fast food dietary pattern was significantly associated with maternal obesity. Metabolic changes caused by obesity in the mother may influence fetal programming and the development of obesity in offspring. Animal studies have shown that prenatal overnutrition (e.g., high-fat diet) results in substantial changes in the development of the central appetitive structures, mainly the hypothalamic neural network [45,46], as well as epigenetic changes in adiponectin and leptin gene expression in offspring which are both adipocytokines and influence insulin sensitivity and the development of metabolic diseases [47]. It also has been reported that a maternal “junk food” diet during pregnancy promotes an exacerbated taste for junk food and leads to a greater propensity for obesity in offspring [48,49]. 

Our study also has several strengths. First, the longitudinal birth cohort with repeated anthropometric measure in children enabled us to prospectively examine the effects of maternal dietary patterns during pregnancy on child growth trajectories, which have been shown to be more predictive for obesity later in life than a single growth measurement [50]. In addition, no previous studies were able to examine the associations between dietary patterns and child growth trajectories. Second, a validated FFQ was used to assess maternal dietary intakes during early pregnancy and enabled dietary pattern analysis. Third, child diet and nutrition change dramatically during infancy and early childhood. Although we could not evaluate children’s dietary patterns from birth to age four, the trajectory analysis of energy intake using the repeated 24-h dietary records collected from the CANDLE children enabled a comprehensive assessment of childhood energy intake over time. This allowed the control for children’s dietary factors to a certain extent when testing the effects of prenatal dietary patterns on childhood fast growth and obesity risk. Finally, the inclusion of both black and white women enabled the generalization of study findings to understudied populations as well as the examination of potential racial disparities in the associations of interest. However, our study did have some limitations. We only conducted the FFQ once during pregnancy, which may not capture the changes in dietary intakes during the entire pregnancy. In addition, we did not collect mothers’ physical activity during pregnancy, which may be another confounding factor. However, the variation of physical activity is very likely to decrease because of reduced physical activity during pregnancy, which may diminish its confounding effect [51,52,53].

## 5. Conclusions

In conclusion, we identified a maternal fast food dietary pattern associated with increased risk for early childhood rapid weight gain and overweight/obesity risk in both black and white populations. Reducing fried food and sugar-sweetened beverage intake during pregnancy may provide an effective approach for reducing the continuing high burden of childhood obesity in the US.

## Figures and Tables

**Table 1:** Factor loadings of major food groups associated with the fast food and processed food patterns ^a^.

Food Group	Fast Food Pattern	Processed Food Pattern
Fried chicken and fish	0.45	
Fruit juices	0.22	
Mayonnaise (as well as margarine and butter)	0.21	
Sugar-sweetened beverages	0.21	
Cold breakfast cereal		0.26
Dairy		0.38
Salad dressing		0.35
Processed meat		0.31
Canned fruit		0.25
French fries		0.24

^a^ Food groups with a factor loading ≥0.2 are considered to have a strong positive association with a dietary pattern.

**Table 2:** Characteristics of the CANDLE mothers and children overall and according to quartiles of the fast food pattern score.

Characteristic	All	Q1 (−5.3~−0.5)	Q2 (−0.5~0)	Q3 (0~0.6)	Q4 (0.6~5.0)	*p* Value ^a^
*n*	1257	314	314	314	315	
**Maternal**						
Age, years	26.3 ± 5.4	26.4 ± 5.8	26.3 ± 5.2	26.5 ± 5.3	25.9 ± 5.3	0.33
Black, %	65.2	59.9	51.9	66.6	82.5	<0.001
Education (≤12 years), %	57.6	56.7	51.9	52.4	69.2	0.002
Marital status (single), %	41.2	37.9	34.7	40.8	51.4	<0.001
Insurance (Medicaid or Medicare), %	57.0	52.9	50.3	53.8	70.8	<0.001
Smoking during pregnancy, %	9.2	7.6	11.8	8.6	8.9	0.94
Alcohol drinking during pregnancy, %	8.8	13.7	7.0	5.8	8.6	0.02
Parity (primiparous), %	30.8	29.9	39.2	31.2	22.9	0.01
Pre-pregnancy BMI, kg/m^2^	27.5 ± 7.5	25.4 ± 6.2	26.6 ± 6.4	28.0 ± 7.3	30.2 ± 8.9	<0.001
Gestational weight gain, kg	14.7 ± 7.3	14.9 ± 6.8	14.6 ± 6.7	14.9 ± 8.2	14.3 ± 7.5	0.41
Gestational diabetes, %	5.7	4.8	5.1	7.1	5.8	0.41
Total energy intake, kcals	2726.4 ± 1666.5	3195.7 ± 1985.6	2223.0 ± 1177.3	2357.2 ± 1484.8	3128.4 ± 1681.6	0.88
Gestational age at enrollment, weeks	23.1 ± 3.0	23.3 ± 3.0	23.1 ± 3.1	23.0 ± 3.1	23.1 ± 2.9	0.52
**Child**						
Gestational age at birth, weeks	38.7 ± 1.9	38.7 ± 2.0	38.8 ± 1.9	38.7 ± 1.9	38.7 ± 1.9	0.79
Male, %	50.8	48.4	49.7	54.8	50.5	0.37
Birth weight, kg	3.2 ± 0.6	3.2 ± 0.5	3.3 ± 0.6	3.2 ± 0.6	3.2 ± 0.6	0.53
Birth length, cm	50.1 ± 3.1	50 ± 3.2	50.4 ± 3.0	50.1 ± 3.0	50.1 ± 3.0	0.81
Breastfed, %	66.6	71.5	66.5	68.6	59.9	0.01
BMIz at birth	−0.4 ± 1.1	−0.4 ± 1.0	−0.4 ± 1.0	−0.4 ± 1.1	−0.5 ± 1.1	0.22
BMIz at age 1	0.7 ± 1.1	0.7 ± 1.1	0.7 ± 1.1	0.8 ± 1.1	0.8 ± 1.2	0.26
BMIz at age 2	0.3 ± 1.2	0.2 ± 1.1	0.3 ± 1.3	0.4 ± 1.3	0.2 ± 1.2	0.67
BMIz at age 3	0.3 ± 1.3	0.3 ± 1.2	0.3 ± 1.3	0.3 ± 1.2	0.3 ± 1.4	0.74
BMIz at age 4	0.5 ± 1.1	0.4 ± 1.1	0.5 ± 1.0	0.6 ± 1.2	0.6 ± 1.3	0.04
Rising-high BMI trajectory, %	12.6	8.6	13.1	13.7	14.9	0.02
Overweight at age 4, %	14.1	12.7	14.3	12.0	16.9	0.29
Obesity at age 4, %	16.4	12.7	13.4	22.2	17.7	0.03
Overweight/obesity at age 4, %	30.5	25.4	27.7	34.3	34.5	0.01

BMI, body mass index; BMIz, sex-specific BMI-for-age z score. ^a^ *P* For trend tests across quartiles.

**Table 3:** Associations between maternal fast food pattern and processed food pattern scores during pregnancy and children’s growth trajectories and overweight/obesity risk at age 4.

Childhood Outcome	Fast Food Pattern			Processed Food Pattern		
	OR (95% CI) ^a^	*p* Value	*p_int_* Value ^b^	OR (95% CI) ^a^	*p* Value	*p_int_* Value^b^
**Model 1 ^c^**						
Low vs. moderate BMI trajectory	1.04 (0.91–1.19)	0.58	0.06	0.91 (0.78–1.06)	0.21	0.82
Rising-high vs. moderate BMI trajectory	1.31 (1.08–1.58)	0.006	0.85	1.04 (0.85–1.28)	0.68	0.98
Overweight vs. normal weight	1.27 (1.04–1.55)	0.02	0.37	1.08 (0.88–1.33)	0.47	0.76
Obesity vs. normal weight	1.24 (1.03–1.50)	0.03	0.53	1.12 (0.93–1.36)	0.23	0.61
Overweight/obesity vs. normal weight	1.25 (1.08–1.46)	0.004	0.89	1.10 (0.94–1.28)	0.23	0.90
**Model 2 ^d^**						
Low vs. moderate BMI trajectory	1.02 (0.88–1.19)	0.79	0.09	1.05 (0.88–1.26)	0.58	0.70
Rising-high vs. moderate BMI trajectory	1.32 (1.07–1.62)	0.008	0.70	0.99 (0.80–1.23)	0.91	0.89
Overweight vs. normal weight	1.31 (1.06–1.61)	0.01	0.23	1.01 (0.81–1.26)	0.92	0.75
Obesity vs. normal weight	1.30 (1.06–1.61)	0.01	0.82	1.08 (0.88–1.33)	0.47	0.48
Overweight/obesity vs. normal weight	1.31 (1.11–1.54)	0.001	0.52	1.04 (0.88–1.24)	0.61	0.80

BMI, body mass index; SE, standard error. ^a^ Associated with a unit increase in the dietary pattern score. ^b^ *P* value for the interaction between the dietary pattern score and maternal race. ^c^ Adjusted for maternal age, race, education, insurance type, marital status, total energy intake, alcohol intake and smoking during pregnancy, parity, and child sex. ^d^ Further adjusted for gestational diabetes, gestational age at birth, birthweight, breastfed status, and childhood energy intake trajectory in addition to the covariables included in Model 1.

## Supplementary Materials

The following are available online at https://www.mdpi.com/2072-6643/12/2/465/s1, Figure S1: BMI-z-score trajectories among the CANDLE children; Figure S2: Early childhood energy intake trajectories among the children of black and white mothers; Supplemental Table S1: Food items within food group; Table S2: Mothers’ eating frequencies of food groups in a month according to quartiles of the fast food pattern score; Table S3: Mothers’ eating frequencies of food groups in a month according to quartiles of the processed food pattern score; Table S4: Mediation analysis of potential mediators for the associations between the maternal fast food pattern and the childhood rising-high-BMI trajectory and overweight/obesity risk at age 4; Table S5: Select nutrient intakes (per 1000 kcal) according to quartiles of the fast food pattern score.

## References

[1] Fryar C.D., Carroll M.D., Ogden C.L. Prevalence of Overweight, Obesity, and Severe Obesity Among Children and Adolescents Aged 2–19 Years: United States, 1963–1965 Through 2015–2016.National Center for health statistics, September 2018. https://www.cdc.gov/nchs/data/hestat/obesity_child_15_16/obesity_child_15_16.htm.

[2] Singh A.S., Mulder C., Twisk J.W., van Mechelen W., Chinapaw M.J. (2008). Tracking of childhood overweight into adulthood: A systematic review of the literature. Obes. Rev..

[3] Brisbois T.D., Farmer A.P., McCargar L.J. (2012). Early markers of adult obesity: A review. Obes. Rev..

[4] Lakshman R., Elks C.E., Ong K.K. (2012). Childhood obesity. Circulation.

[5] Dabelea D., Harrod C.S. (2013). Role of developmental overnutrition in pediatric obesity and type 2 diabetes. Nutr. Rev..

[6] Juonala M., Magnussen C.G., Berenson G.S., Venn A., Burns T.L., Sabin M.A., Srinivasan S.R., Daniels S.R., Davis P.H., Chen W. (2011). Childhood adiposity, adult adiposity, and cardiovascular risk factors. N. Engl. J. Med..

[7] Baker J.L., Olsen L.W., Sorensen T.I. (2007). Childhood body-mass index and the risk of coronary heart disease in adulthood. N. Engl. J. Med..

[8] Umer A., Kelley G.A., Cottrell L.E., Giacobbi P., Innes K.E., Lilly C.L. (2017). Childhood obesity and adult cardiovascular disease risk factors: A systematic review with meta-analysis. BMC Public Health.

[9] Luca P., Birken C., Grewal P., Dettmer E., Hamilton J. (2012). Complex Obesity. Curr. Pediatr. Rev..

[10] Hammond R.A., Levine R. (2010). The economic impact of obesity in the United States. Diabetes Metab. Syndr. Obes..

[11] Cawley J. (2010). The economics of childhood obesity. Health Aff..

[12] Trasande L., Chatterjee S. (2009). The impact of obesity on health service utilization and costs in childhood. Obesity.

[13] Heindel J.J., Vandenberg L.N. (2015). Developmental origins of health and disease: A paradigm for understanding disease cause and prevention. Curr. Opin. Pediatr..

[14] Skinner A.C., Steiner M.J., Henderson F.W., Perrin E.M. (2010). Multiple markers of inflammation and weight status: Cross-sectional analyses throughout childhood. Pediatrics.

[15] Wu G., Bazer F.W., Cudd T.A., Meininger C.J., Spencer T.E. (2004). Maternal nutrition and fetal development. J. Nutr..

[16] Zhu Y., Olsen S.F., Mendola P., Halldorsson T.I., Yeung E.H., Granstrom C., Bjerregaard A.A., Wu J., Rawal S., Chavarro J.E. (2017). Maternal dietary intakes of refined grains during pregnancy and growth through the first 7 y of life among children born to women with gestational diabetes. Am. J. Clin. Nutr..

[17] Moon R.J., Harvey N.C., Robinson S.M., Ntani G., Davies J.H., Inskip H.M., Godfrey K.M., Dennison E.M., Calder P.C., Cooper C. (2013). Maternal plasma polyunsaturated fatty acid status in late pregnancy is associated with offspring body composition in childhood. J. Clin. Endocrinol. Metab..

[18] Hu F.B. (2002). Dietary pattern analysis: A new direction in nutritional epidemiology. Curr. Opin. Lipidol..

[19] van den Broek M., Leermakers E.T., Jaddoe V.W., Steegers E.A., Rivadeneira F., Raat H., Hofman A., Franco O.H., Kiefte-de Jong J.C. (2015). Maternal dietary patterns during pregnancy and body composition of the child at age 6 y: The Generation R Study. Am. J. Clin. Nutr..

[20] Murrin C.M., Heinen M.M., Kelleher C.C. (2015). Are Dietary Patterns of Mothers during Pregnancy Related to Children’s Weight Status? Evidence from the Lifeways Cross- Generational Cohort Study. AIMS Public Health.

[21] Martin C.L., Siega-Riz A.M., Sotres-Alvarez D., Robinson W.R., Daniels J.L., Perrin E.M., Stuebe A.M. (2016). Maternal Dietary Patterns during Pregnancy Are Associated with Child Growth in the First 3 Years of Life. J. Nutr..

[22] Chen L.W., Aris I.M., Bernard J.Y., Tint M.T., Chia A., Colega M., Gluckman P.D., Shek L.P., Saw S.M., Chong Y.S. (2016). Associations of Maternal Dietary Patterns during Pregnancy with Offspring Adiposity from Birth Until 54 Months of Age. Nutrients.

[23] Palmer F.B., Anand K.J., Graff J.C., Murphy L.E., Qu Y., Volgyi E., Rovnaghi C.R., Moore A., Tran Q.T., Tylavsky F.A. (2013). Early adversity, socioemotional development, and stress in urban 1-year-old children. J. Pediatr..

[24] Tylavsky F.A., Kocak M., Murphy L.E., Graff J.C., Palmer F.B., Volgyi E., Diaz-Thomas A.M., Ferry R.J. (2015). Gestational Vitamin 25(OH)D Status as a Risk Factor for Receptive Language Development: A 24-Month, Longitudinal, Observational Study. Nutrients.

[25] Volgyi E., Carroll K.N., Hare M.E., Ringwald-Smith K., Piyathilake C., Yoo W., Tylavsky F.A. (2013). Dietary patterns in pregnancy and effects on nutrient intake in the Mid-South: The Conditions Affecting Neurocognitive Development and Learning in Early Childhood (CANDLE) study. Nutrients.

[26] Subar A.F., Thompson F.E., Kipnis V., Midthune D., Hurwitz P., McNutt S., McIntosh A., Rosenfeld S. (2001). Comparative validation of the Block, Willett, and National Cancer Institute food frequency questionnaires: The Eating at America’s Table Study. Am. J. Epidemiol..

[27] Hoffmann K., Schulze M.B., Schienkiewitz A., Nothlings U., Boeing H. (2004). Application of a new statistical method to derive dietary patterns in nutritional epidemiology. Am. J. Epidemiol..

[28] Johnson L., Mander A.P., Jones L.R., Emmett P.M., Jebb S.A. (2008). Energy-dense, low-fiber, high-fat dietary pattern is associated with increased fatness in childhood. Am. J. Clin. Nutr..

[29] Hu Z., Tylavsky F.A., Han J.C., Kocak M., Fowke J.H., Davis R.L., Lewinn K., Bush N.R., Zhao Q. (2019). Maternal metabolic factors during pregnancy predict early childhood growth trajectories and obesity risk: The CANDLE Study. Int. J. Obes..

[30] Sridhar S.B., Darbinian J., Ehrlich S.F., Markman M.A., Gunderson E.P., Ferrara A., Hedderson M.M. (2014). Maternal gestational weight gain and offspring risk for childhood overweight or obesity. Am. J. Obstet. Gynecol..

[31] Kaseva N., Vaarasmaki M., Matinolli H.M., Sipola-Leppanen M., Tikanmaki M., Heinonen K., Lano A., Wolke D., Andersson S., Jarvelin M.R. (2018). Pre-pregnancy overweight or obesity and gestational diabetes as predictors of body composition in offspring twenty years later: Evidence from two birth cohort studies. Int. J. Obes..

[32] Lau E.Y., Liu J., Archer E., McDonald S.M., Liu J. (2014). Maternal weight gain in pregnancy and risk of obesity among offspring: A systematic review. J. Obes..

[33] Vermeulen E., Stronks K., Visser M., Brouwer I.A., Snijder M.B., Mocking R.J.T., Derks E.M., Schene A.H., Nicolaou M. (2017). Dietary pattern derived by reduced rank regression and depressive symptoms in a multi-ethnic population: The HELIUS study. Eur. J. Clin. Nutr..

[34] Batis C., Mendez M.A., Gordon-Larsen P., Sotres-Alvarez D., Adair L., Popkin B. (2016). Using both principal component analysis and reduced rank regression to study dietary patterns and diabetes in Chinese adults. Public Health Nutr..

[35] de Jonge E.A., Kiefte-de Jong J.C., Hofman A., Uitterlinden A.G., Kieboom B.C., Voortman T., Franco O.H., Rivadeneira F. (2017). Dietary patterns explaining differences in bone mineral density and hip structure in the elderly: The Rotterdam Study. Am. J. Clin. Nutr..

[36] Schulze M.B., Hoffmann K., Kroke A., Boeing H. (2003). An approach to construct simplified measures of dietary patterns from exploratory factor analysis. Br. J. Nutr..

[37] Colon-Ramos U., Racette S.B., Ganiban J., Nguyen T.G., Kocak M., Carroll K.N., Volgyi E., Tylavsky F.A. (2015). Association between dietary patterns during pregnancy and birth size measures in a diverse population in Southern US. Nutrients.

[38] Fernandez-Barres S., Romaguera D., Valvi D., Martinez D., Vioque J., Navarrete-Munoz E.M., Amiano P., Gonzalez-Palacios S., Guxens M., Pereda E. (2016). Mediterranean dietary pattern in pregnant women and offspring risk of overweight and abdominal obesity in early childhood: The INMA birth cohort study. Pediatr. Obes..

[39] Grummer-Strawn L.M., Reinold C., Krebs N.F. (2010). Use of World Health Organization and CDC growth charts for children aged 0–59 months in the United States. MMWR Recomm. Rep..

[40] Morrison J., Regnault T. (2016). Nutrition in Pregnancy: Optimising Maternal Diet and Fetal Adaptations to Altered Nutrient Supply. Nutrients.

[41] Imai K., Keele L., Yamamoto T. (2010). Identification, inference, and sensitivity analysis for causal mediation effects. Stat. Sci..

[42] Osorio-Yáñez C., Gelaye B., Qiu C., Bao W., Cardenas A., Enquobahrie D.A., Williams M.A. (2017). Maternal intake of fried foods and risk of gestational diabetes mellitus. Ann. Epidemiol..

[43] Jen V., Erler N.S., Tielemans M.J., Braun K.V., Jaddoe V.W., Franco O.H., Voortman T. (2017). Mothers’ intake of sugar-containing beverages during pregnancy and body composition of their children during childhood: The Generation R Study. Am. J. Clin. Nutr..

[44] Kim S.Y., Sharma A.J., Callaghan W.M. (2012). Gestational diabetes and childhood obesity: What is the link?. Curr. Opin. Obstet. Gynecol..

[45] McMillen I.C., Adam C.L., Muhlhausler B.S. (2005). Early origins of obesity: Programming the appetite regulatory system. J. Physiol..

[46] Plagemann A., Harder T., Janert U., Rake A., Rittel F., Rohde W., Dorner G. (1999). Malformations of hypothalamic nuclei in hyperinsulinemic offspring of rats with gestational diabetes. Dev. Neurosci..

[47] Masuyama H., Mitsui T., Nobumoto E., Hiramatsu Y. (2015). The Effects of High-Fat Diet Exposure In Utero on the Obesogenic and Diabetogenic Traits Through Epigenetic Changes in Adiponectin and Leptin Gene Expression for Multiple Generations in Female Mice. Endocrinology.

[48] Bayol S.A., Farrington S.J., Stickland N.C. (2007). A maternal ‘junk food’ diet in pregnancy and lactation promotes an exacerbated taste for ‘junk food’ and a greater propensity for obesity in rat offspring. Br. J. Nutr..

[49] Erlanson-Albertsson C. (2005). How palatable food disrupts appetite regulation. Basic Clin. Pharmacol. Toxicol..

[50] Aris I.M., Chen L.W., Tint M.T. (2017). Body mass index trajectories in the first two years and subsequent childhood cardio-metabolic outcomes: A prospective multi-ethnic Asian cohort study. Sci. Rep..

[51] Haakstad L.A., Voldner N., Henriksen T., Bo K. (2007). Physical activity level and weight gain in a cohort of pregnant Norwegian women. Acta Obstet. Gynecol. Scand..

[52] Pereira M.A., Rifas-Shiman S.L., Kleinman K.P., Rich-Edwards J.W., Peterson K.E., Gillman M.W. (2007). Predictors of change in physical activity during and after pregnancy: Project Viva. Am. J. Prev. Med..

[53] Coll C., Domingues M., Santos I., Matijasevich A., Horta B.L., Hallal P.C. (2016). Changes in Leisure-Time Physical Activity From the Prepregnancy to the Postpartum Period: 2004 Pelotas (Brazil) Birth Cohort Study. J. Phys. Act. Health.

