# Supplementary material for: Effects of Maternal Dietary Patterns during Pregnancy on Early Childhood Growth Trajectories and Obesity Risk: The CANDLE Study
Source: Nutrients. 2020 Feb 13;12(2):465. doi: 10.3390/nu12020465 (PMC7071328; doi:10.3390/nu12020465)
Supplement: Supplementary file 1 [file nutrients-12-00465-s001.pdf]

Supplemental Figure 1. BMI-z-score trajectories among the CANDLE children.

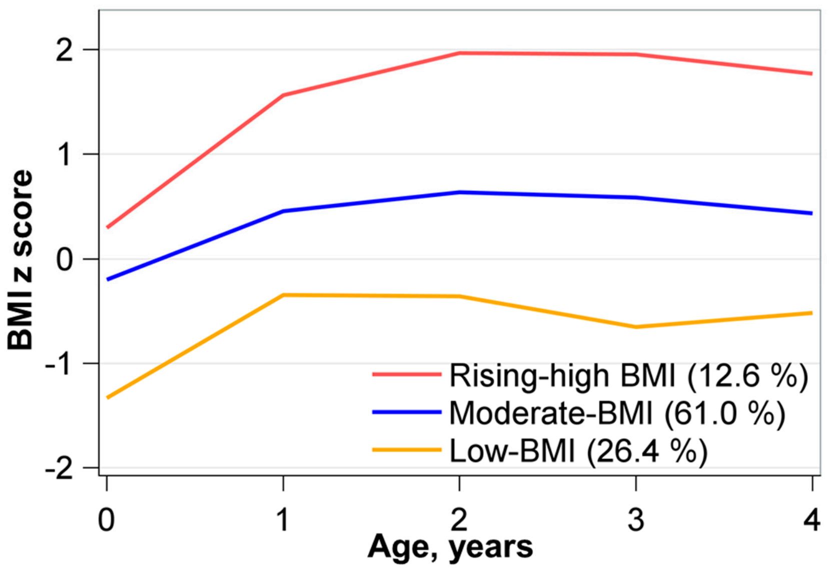

Supplemental Figure 2. Early childhood energy intake trajectories among the children of black and white mothers.

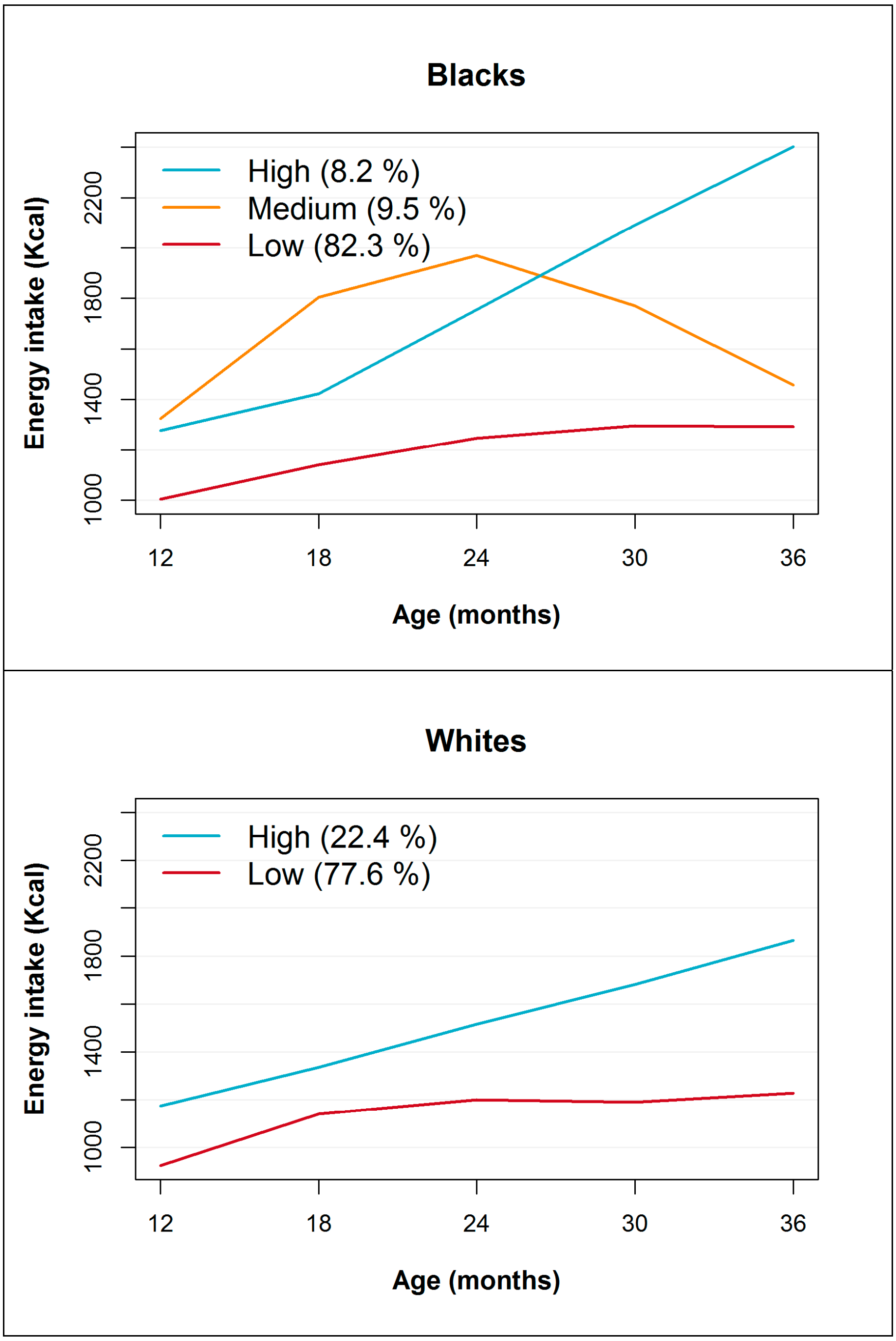

Supplemental Table 1. Food items within each food group.

Food groups	Food items
Red meat	Beef, pork, ribs, veal, meat loaf, and hamburger (cheeseburger)
Processed meat	Breakfast sausage, bacon, hot dogs, and lunch meats
Organ meat	Liver, pig feet, and menudo
Poultry	Roast chicken
Mixed meat	Tacos, other beef dishes, and other chicken dishes
Fish and other seafood	Oysters, shellfish, tuna, and other fish
Fried chicken and fish	Fried chicken and fried fish
Egg	Breakfast egg sandwich and other eggs
Dairy	Milk, milk on cereal, yogurt, cheese, ice cream, and Slim Fast
Sugar-sweetened beverages	Hi-C, drinks with some juice, iced tea, Kool-Aid, sports drinks, and soft drink
Fruit juices	Real orange juice and other real juice
Alcohol	Beer, wine, and liquor
Tea and Coffee	Coffee and hot tea
Water	Water
Fruit	Bananas, apples, pears, oranges, tangerines, grapefruits, peaches, cantaloupes, strawberries, watermelons, and other fresh fruits
Canned fruit	Canned fruit
Greens	Greens
Spinach	Spinach
Tomatoes	Tomatoes and tomato juice
Yellow vegetables	Carrots and sweet potato
Potatoes	Potatoes
French fries	French fries
Other vegetables	Broccoli, corn, green beans, cole slaw, vegetable stew, vegetable soup, and other vegetables
Legumes	Refried beans, bean soup, and other beans
Tofu and meat substitutes	Tofu and meat substitutes
Mayonnaise, margarine and butter	Margarine, butter and mayonnaise
Salad dressing	Green salad and salad dressing
Condiments	Jelly, catsup, and mustard
Whole grains	Cooked cereal
Cold breakfast cereal	Cold cereal
Bread products	Pancakes, biscuits, burger rolls, bagels, tortillas (flour), corn bread, and white bread
Pizza	Pizza
Rice, pasta, mixed and dishes	Spaghetti with meat sauce, macaroni and cheese, rice, other soup, and other noodles
Nuts	Nuts and peanut butter
Sweets and desserts	Donuts, cakes, cookies, chocolate syrup, pumpkin pie, chocolate candy, other pie, and other candy
Snacks	Power bars, breakfast bars, chips, and crackers

Supplemental Table 2. Mothers' eating frequencies of food groups in a month according to quartiles of the fast food pattern score.

Food group	Q1	Q2	Q3	Q4	*P* value[a]
Red meat	13.2 ± 14.0	9.5 ± 9.0	10.1 ± 8.7	14.7 ± 13.5	0.08
Processed meat	22.7 ± 19.7	17.0 ± 15.8	18.5 ± 16.2	24.8 ± 18.5	0.08
Organ meats	0.9 ± 3.2	0.5 ± 1.4	0.4 ± 1.3	1.0 ± 2.4	0.79
Poultry	4.6 ± 6.1	3.6 ± 4.1	3.9 ± 5.4	4.1 ± 5.1	0.35
Mixed meat	7.5 ± 8.8	5.4 ± 4.6	6.4 ± 6.4	7.3 ± 8.1	0.75
Fish and other seafood	3.4 ± 4.2	3.3 ± 3.9	3.1 ± 3.6	4.4 ± 6.2	0.02
Fried chicken and fish	6.1 ± 7.6	4.7 ± 5.7	6.2 ± 6.7	13.1 ± 10.6	< 0.001
Egg	10.0 ± 11.5	8.9 ± 9.7	9.9 ± 10.0	13.9 ± 12.6	< 0.001
Dairy	59.7 ± 28.2	52.8 ± 25.4	49.6 ± 24.6	54.1 ± 27.0	0.002
Sugar-sweetened beverages	32.5 ± 26.7	25.2 ± 19.6	27.6 ± 20.9	41.1 ± 28.7	< 0.001
Fruit juices	17.3 ± 15.9	16.8 ± 14.0	19.4 ± 15.0	25.1 ± 18.3	< 0.001
Alcohol	0.3 ± 1.6	0.1 ± 0.5	0.0 ± 0.3	0.0 ± 0.2	< 0.001
Tea and coffee	8.2 ± 13.1	4.5 ± 9.5	2.2 ± 6.1	1.5 ± 4.7	< 0.001
Water	24.9 ± 9.3	26.1 ± 8.5	27.8 ± 6.4	27.6 ± 6.9	< 0.001
Fruit	47.1 ± 35.3	39.3 ± 28.5	40.1 ± 29.4	51.8 ± 37.0	0.07
Canned fruit	6.4 ± 8.9	5.0 ± 7.0	5.9 ± 8.0	9.4 ± 9.8	< 0.001
Greens	2.6 ± 4.7	1.9 ± 4.0	2.3 ± 4.2	3.4 ± 5.2	0.008
Spinach	1.7 ± 3.9	1.6 ± 3.9	1.4 ± 3.2	2.1 ± 4.8	0.31
Tomatoes	6.1 ± 9.3	6.2 ± 8.2	5.7 ± 8.3	7.7 ± 9.8	0.046
Yellow vegetables	9.1 ± 10.5	5.4 ± 6.3	4.4 ± 5.2	4.2 ± 5.6	< 0.001
Potatoes	6.1 ± 6.6	4.6 ± 4.4	5.0 ± 5.7	6.7 ± 6.9	0.16
French fries	9.7 ± 9.3	7.1 ± 7.4	7.1 ± 7.4	10.2 ± 9.1	0.46
Other vegetables	23.6 ± 20.9	21.0 ± 16.6	21.1 ± 16.0	28.5 ± 22.7	0.002
Legumes	5.1 ± 8.0	3.6 ± 4.3	3.1 ± 3.6	3.6 ± 5.6	< 0.001
Tofu and meat substitutes	1.0 ± 3.7	0.5 ± 1.9	0.3 ± 1.5	0.3 ± 2.1	< 0.001
Mayonnaise, margarine and butter	17.8 ± 15.8	15.7 ± 14.6	17.2 ± 16.1	23.6 ± 18.7	< 0.001
Salad dressing	16.8 ± 15.6	15.0 ± 13.8	15.9 ± 15.7	19.6 ± 16.6	0.02
Condiments	20.7 ± 16.6	16.2 ± 13.4	18.3 ± 16.0	23.4 ± 18.6	0.01
Whole grains	7.5 ± 9.8	5.1 ± 7.1	4.6 ± 7.1	5.5 ± 8.4	0.002
Cold breakfast cereal	17.4 ± 10.9	14.8 ± 10.1	11.8 ± 10.2	12.4 ± 10.7	< 0.001
Bread products	42.3 ± 26.8	32.7 ± 20.3	32.2 ± 23.2	39.3 ± 23.0	0.11
Pizza	5.8 ± 6.9	3.8 ± 4.3	3.7 ± 4.5	4.3 ± 4.9	< 0.001
Rice, pasta, mixed, dishes	22.6 ± 17.3	14.5 ± 10.3	14.1 ± 12.0	16.4 ± 13.3	< 0.001
Nuts	9.9 ± 11.0	7.7 ± 8.8	5.8 ± 7.8	6.1 ± 9.2	< 0.001
Sweets and desserts	31.4 ± 30.3	19.5 ± 22.2	18.0 ± 19.4	22.2 ± 24.0	< 0.001
Snacks	29.8 ± 19.5	21.9 ± 16.1	19.1 ± 16.8	21.1 ± 16.5	< 0.001

[a.] For trend tests.

Supplemental Table 3. Mothers' eating frequencies of food groups in a month according to quartiles of the processed food pattern score.

Food group	Q1	Q2	Q3	Q4	*P* value[a]
Red meat	13.5 ± 14.4	9.8 ± 8.4	10.8 ± 10.2	13.5 ± 12.6	0.78
Processed meat	19.3 ± 17.7	16.5 ± 14.9	18.2 ± 14.0	29.1 ± 21.3	< 0.001
Organ meat	1.5 ± 3.7	0.4 ± 1.1	0.3 ± 0.8	0.6 ± 1.9	< 0.001
Poultry	3.9 ± 5.4	3.9 ± 5.3	4.2 ± 5.3	4.0 ± 4.8	0.64
Mixed meat	7.4 ± 8.5	6.3 ± 6.5	5.8 ± 5.3	7.0 ± 7.9	0.31
Fish and other seafood	4.7 ± 6.2	3.6 ± 3.9	3.0 ± 4.1	2.9 ± 3.5	< 0.001
Fried chicken and fish	9.4 ± 10.7	7.0 ± 7.7	5.5 ± 5.8	8.4 ± 8.7	0.03
Egg	9.9 ± 11.9	9.2 ± 9.9	9.4 ± 9.3	14.3 ± 12.6	< 0.001
Dairy	44.8 ± 25.0	47.1 ± 22.0	56.6 ± 25.8	67.8 ± 26.8	< 0.001
Sugar-sweetened beverages	43.6 ± 28.6	28.6 ± 23.5	26.4 ± 20.4	27.8 ± 23.1	< 0.001
Fruit juices	18.8 ± 16.4	17.6 ± 15.5	18.4 ± 14.8	23.8 ± 17.4	< 0.001
Alcohol	0.2 ± 1.4	0.1 ± 0.7	0.1 ± 0.6	0.1 ± 0.5	0.04
Tea and Coffee	6.4 ± 11.9	4.9 ± 10.0	3.2 ± 7.7	1.9 ± 6.1	< 0.001
Water	25.4 ± 9.1	26.2 ± 8.3	27.0 ± 7.6	27.8 ± 6.6	< 0.001
Fruit	51.1 ± 35.6	42.5 ± 31.3	38.8 ± 28.7	45.9 ± 35.2	0.02
Canned fruit	5.1 ± 7.4	5.0 ± 7.0	6.2 ± 7.9	10.4 ± 10.7	< 0.001
Greens	2.8 ± 4.8	1.9 ± 3.9	1.9 ± 3.3	3.7 ± 5.7	0.04
Spinach	1.3 ± 3.2	1.4 ± 3.1	1.4 ± 3.5	2.5 ± 5.6	< 0.001
Tomatoes	6.4 ± 9.7	5.5 ± 7.5	5.8 ± 7.6	8.0 ± 10.5	0.023
Yellow vegetables	5.4 ± 7.5	5.3 ± 6.7	4.7 ± 5.9	7.7 ± 9.0	< 0.001
Potatoes	6.0 ± 6.0	4.5 ± 5.1	5.1 ± 5.4	6.9 ± 7.3	0.03
French fries	8.3 ± 8.4	6.7 ± 6.6	7.7 ± 7.3	11.3 ± 10.3	< 0.001
Other vegetables	22.7 ± 18.0	20.9 ± 17.0	20.3 ± 16.0	30.3 ± 24.3	< 0.001
Legumes	5.6 ± 7.8	3.4 ± 4.4	3.1 ± 3.6	3.2 ± 5.7	< 0.001
Tofu and meat substitutes	0.5 ± 1.8	0.6 ± 2.6	0.5 ± 1.9	0.5 ± 3.3	1.00
Mayonnaise, margarine and butter	18.6 ± 17.9	16.3 ± 14.5	16.9 ± 15.2	22.6 ± 17.9	0.002
Salad dressing	12.2 ± 13.0	13.6 ± 12.9	16.7 ± 13.5	24.7 ± 18.9	< 0.001
Condiments	22.5 ± 19.1	18.2 ± 14.4	18.5 ± 15.3	19.6 ± 16.4	0.03
Whole grains	4.4 ± 7.5	4.8 ± 7.0	5.4 ± 7.6	8.2 ± 10.0	< 0.001
Cold breakfast cereal	11.5 ± 10.5	12.5 ± 10.0	14.7 ± 10.4	17.7 ± 11.0	< 0.001
Bread products	41.6 ± 28.8	33.0 ± 20.9	32.6 ± 20.1	39.3 ± 23.1	0.23
Pizza	4.5 ± 5.5	4.2 ± 4.9	4.0 ± 4.6	4.9 ± 6.0	0.55
Rice, pasta, mixed and dishes	18.4 ± 15.2	15.7 ± 12.7	15.1 ± 12.2	18.5 ± 14.9	0.87
Nuts	8.5 ± 10.2	7.0 ± 9.0	6.8 ± 8.6	7.2 ± 9.6	0.09
Sweets and desserts	29.2 ± 31.1	20.9 ± 20.9	19.7 ± 21.2	21.3 ± 23.6	< 0.001
Snacks	26.0 ± 19.2	20.8 ± 15.6	20.6 ± 15.6	24.4 ± 19.6	0.24

[a.] For trend tests.

Supplemental Table 4. Mediation analysis of potential mediators for the associations between the maternal fast food pattern and the childhood rising-high-BMI trajectory and overweight/obesity risk at age 4[a]

Mediator	Rising-high-BMI trajectory		Overweight/obesity at age 4	
	**Mediation effect**	***P* value**	**Mediation effect**	***P* value**
Gestational diabetes	0.0000	0.93	-0.0001	1.00
Gestational age at birth	-0.0007	0.42	-0.0023	0.09
Birthweight	-0.0015	0.50	-0.0013	0.61
Breastfed status	-0.0004	0.62	-0.0007	0.60
Childhood energy intake trajectory	0.0003	0.76	-0.0004	0.80

[a.] Adjusted for processed dietary pattern score, maternal age, education, insurance type, marital status, total energy intake, alcohol intake, smoking during pregnancy, parity, and child sex.

Supplemental Table 5. Selected nutrient intakes (per 1000 kcal) according to quartiles of the fast food pattern score.

Nutrients	Q1	Q2	Q3	Q4	*P* value[a]
Protein, g	36.4 ± 6.0	37.3 ± 6.2	37.8 ± 6.4	37.6 ± 6.3	0.013
Fat, g	39.8 ± 5.0	40.2 ± 5.8	40.5 ± 5.8	41.5 ± 5.8	< 0.001
Carbohydrate, g	128.8 ± 14.3	127.4 ± 16.6	125.8 ± 16.3	123.4 ± 17.4	< 0.001
Total sugar, g	59.3 ± 14.9	61.5 ± 15.9	63.7 ± 17.7	65.9 ± 18.2	< 0.001
Fiber, g	9.5 ± 3.3	9.4 ± 2.9	8.9 ± 3.0	8.0 ± 2.5	< 0.001
Cholesterol, mg	122.8 ± 42.4	133.2 ± 51.6	140.2 ± 46.6	155.2 ± 60.3	< 0.001
Vitamin A, RAE	436.5 ± 171.9	435.8 ± 162.2	407.6 ± 153.9	396.4 ± 165.0	< 0.001
Vitamin C, mg	63.4 ± 25.7	68.3 ± 29.4	76.4 ± 31.0	80.7 ± 34.6	< 0.001
Vitamin D, IU	75.0 ± 44.0	86.5 ± 58.4	79.0 ± 55.3	69.7 ± 40.5	0.06
Vitamin E, mg	3.9 ± 1.1	3.9 ± 1.2	3.8 ± 1.1	3.7 ± 0.9	0.009
Vitamin K, µg	93.0 ± 67.9	97.4 ± 66.6	101.7 ± 79.5	104.5 ± 79.5	0.037
Beta-cryptoxanthin, µg	91.3 ± 58.7	109.3 ± 67.6	110.7 ± 70.4	113.3 ± 70.3	< 0.001
Lutein-zeaxanthin, µg	1673.8 ± 1312.8	1753.9 ± 1219.3	1826.2 ± 1503.1	1876.6 ± 1487.6	0.05
Lycopene, µg	2681.3 ± 1756.7	2665.8 ± 1997.1	2591.1 ± 1712.5	2393.2 ± 1778.6	0.04
Potassium, mg	1301.0 ± 286.2	1357.7 ± 295.8	1356.1 ± 281.2	1308.0 ± 296.9	0.8
Fe, mg	8.0 ± 1.8	7.7 ± 1.9	7.2 ± 1.7	6.8 ± 1.2	< 0.001
Zn (animal sources), mg	2.7 ± 0.8	2.8 ± 0.9	3.0 ± 0.8	3.1 ± 1.4	< 0.001
Magnesium, mg	145.7 ± 37.4	148.3 ± 34.9	144.8 ± 35.9	132.5 ± 30.8	< 0.001
Total choline, mg	140.7 ± 31.4	148.7 ± 35.0	149.7 ± 33.0	152.1 ± 40.0	< 0.001
Folate equivalents, µg	305.0 ± 91.2	297.1 ± 101.3	274.9 ± 95.2	256.9 ± 74.5	< 0.001
Folic acid, µg	103.3 ± 47.4	94.2 ± 52.7	80.6 ± 41.7	72.8 ± 31.6	< 0.001

IU, international unit. RAE, retinol activity equivalent.

[a.] For trend tests.
